# Chromosome 11q13 amplification as a decision-making biomarker for anti-PD-1 immunotherapy in recurrent or metastatic head and neck squamous cell carcinoma: a prospective cohort study

**DOI:** 10.3389/fimmu.2025.1667733

**Published:** 2025-10-13

**Authors:** Wen Jiang, Shengjin Dou, Lin Zhang, Minjun Dong, Jiang Li, Ge Wang, Ziyue Gu, Yining He, Debin Sun, Rongrong Li, Guopei Zhu

**Affiliations:** ^1^ Department of Oral and Maxillofacial Head & Neck Oncology, Shanghai Ninth People’s Hospital, College of Stomatology, Shanghai Jiao Tong University School of Medicine, Shanghai, China; ^2^ National Clinical Research Center for Oral Diseases, Shanghai, China; ^3^ Shanghai Key Laboratory of Stomatology & Shanghai Research Institute of Stomatology, Shanghai, China; ^4^ Department of Radiology, Shanghai Ninth People’s Hospital, Shanghai Jiao Tong University, School of Medicine, Shanghai, China; ^5^ Department of Oral Pathology, Shanghai Ninth People’s Hospital, Shanghai Jiao Tong University, School of Medicine, Shanghai, China; ^6^ Biostatistics Office of Clinical Research Unit, Shanghai Ninth People’s Hospital, Shanghai Jiao Tong University School of Medicine, Shanghai, China; ^7^ Department of Medicine, Genecast Biotechnology Co., Ltd, Wuxi, China

**Keywords:** recurrent and metastatic head and neck cancer, 11q13 amplification, anti-pd 1 immunotherapy, biomarker, personlised therapy

## Abstract

**Background:**

Anti-programmed cell death protein 1 (anti-PD-1) immunotherapy has shown efficacy in recurrent or metastatic head and neck squamous cell carcinoma (R/M HNSCC), but current biomarkers have limitations in predicting immunotherapy response accurately. Chromosome 11q13 amplification, prevalent in HNSCC, has been associated with reduced efficacy of anti-PD-1 therapy. This study aims to prospectively evaluate 11q13 amplification as a biomarker for guiding first-line treatment in R/M HNSCC. We hypothesize that excluding patients with 11q13 amplification from anti-PD-1 therapy may enhance survival outcomes.

**Methods:**

This single-institution prospective cohort study included previously untreated patients with R/M HNSCC. Based on 11q13 amplification status, non-amplified patients received PD-1 inhibitor monotherapy or combination therapy with chemotherapy, while amplified patients were treated with cetuximab and chemotherapy. Nedaplatin was used in place of cisplatin if necessary. Ten 11q13-amplified patients receiving anti-PD-1 therapy served as an external control group.

**Results:**

Between August 2020 and June 2023, 75 patients were enrolled prospectively, and an additional 10 patients with 11q13 amplification were included as an external control. Among R/M HNSCC patients without 11q13 amplification who received anti-PD-1-based therapy, the objective response rate (ORR) was 72.5%, with a median progression-free survival (PFS) of 14.3 months and an overall survival (OS) of 38.2 months. These survival outcomes were superior to those seen in other cohorts within this study and reported in other trials.

**Conclusions:**

Our study suggests that 11q13 amplification status could serve as a valuable biomarker for first-line treatment decisions in R/M HNSCC. Patients without 11q13 amplification exhibited better responses to anti-PD-1 therapy, providing insights into optimizing treatment strategies.

**Clinical trial registration:**

Chinese Clinical Trial Registry identifier, ChiCTR2000035635.

## Introduction

Head and neck squamous cell carcinoma (HNSCC) remains a significant clinical challenge, with over 65% of patients developing recurrence or metastasis despite advancements in treatment approaches (1, 2). The combination of cetuximab with platinum-based chemotherapy has become the standard first-line treatment strategy following the EXTREME study (3). Recently, the phase III KEYNOTE-048 trial demonstrated durable clinical responses and improved survival outcomes in recurrent or metastatic HNSCC (R/M HNSCC) patients treated with anti-programmed cell death protein 1 (anti-PD-1) therapy, leading to its approval as a first-line treatment for R/M HNSCC (4–6). However, concerns persist regarding the optimal first-line therapeutic strategy incorporating anti-PD-1 therapy (7, 8). In the overall population of the KEYNOTE-048 trial, pembrolizumab monotherapy did not demonstrate superior overall survival (OS) compared to the EXTREME regimen. It was also associated with inferior progression-free survival (PFS) and lower response rates (4).

The combined positive score (CPS) is the most widely used biomarker for assessing the potential benefits of immunotherapy. However, CPS alone is insufficient in predicting response for anti-PD-1 therapy, as over 30% of patients with CPS > 20 treated with pembrolizumab monotherapy experienced progressive disease (PD) (4). Given the limitations of the current biomarker in evaluating treatment efficacy, identifying patients who are more likely to benefit from immunotherapy is an unmet task.

The chromosome 11q13, which includes cyclin D1 (*CCND1*), fibroblast growth factor 3 (*FGF3*), *FGF4*, and *FGF19*, is frequently amplified in HNSCC, with incidences ranging from 20% to 40% (9–13). Our previous retrospective analysis revealed a negative association between 11q13 amplification (Amp11q13) and the efficacy of anti-PD-1 therapy, irrespective of CPS status (14). Furthermore, bioinformatic analyses of The Cancer Genome Atlas (TCGA) and a cohort study revealed that *CCND1* amplification was associated with poor response to immune checkpoint inhibitors (ICIs) (15, 16). Building on these preliminary findings, we conducted a prospective clinical investigation to evaluate the role of 11q13 amplification (Amp11q13) in guiding first-line treatment decisions for R/M HNSCC patients. The aim of this study was to determine whether excluding patients with Amp11q13 from anti-PD-1 therapy could improve objective response rates and survival outcomes. We hypothesized that Amp11q13 amplification could serve as a predictive biomarker to identify patients more likely to benefit from anti-PD-1 therapy.

## Materials and methods

### Study design and patients

This single-institution, prospective exploratory cohort study was conducted at the Shanghai Ninth People’s Hospital. We enrolled previously untreated patients with R/M HNSCC between August 2020 and June 2023. The key inclusion criteria were as follows: 1) age ≥ 18 years; 2) histologically confirmed R/M HNSCC and ineligible for salvage surgery; 3) Eastern Cooperative Oncology Group performance status of 0–1; 4) availability of adequate formalin-fixed paraffin-embedded (FFPE) tissue samples for immunohistochemistry staining and next-generation sequencing (NGS). The first-line treatment regimen was selected based on the presence or absence of Amp11q13. This study was approved by the Shanghai Ninth People’s Hospital Institutional Review Board (registration number: ChiCTR2000035635, ethical approval number: SHS9H-2020-T25). An external control group was established using a retrospective cohort of ten patients with confirmed Amp11q13 amplification. These patients received anti-PD-1 immunotherapy with or without chemotherapy between June 2019 and June 2020. The cohort was selected to enable comparative analysis of treatment outcomes.

### Treatments

Treatment strategies were based on Amp11q13 status. For patients without Amp11q13, the first-line treatment was a PD-1 inhibitor, either as monotherapy or in combination with chemotherapy. The chemotherapy regimens included paclitaxel-albumin (220 mg/m², every 3 weeks [q3w]) combined with cisplatin (75 mg/m², q3w) or docetaxel (75 mg/m², q3w) with cisplatin (75 mg/m², q3w). As this was a cohort study, no specific anti-PD-1 agent was mandated; the anti-PD-1 agents used included pembrolizumab (200 mg), tislelizumab (200 mg), camrelizumab (200 mg), and toripalimab (240 mg), the anti-PD-1 agent was administered q3w. Patients with 11q13 amplification received cetuximab (400 mg/m² loading dose, followed by 250 mg/m² weekly) in combination with paclitaxel-albumin and cisplatin (or docetaxel and cisplatin), with chemotherapy dosages and administration as described above. For patients who are intolerant to cisplatin, nedaplatin was substituted at the treating physician’s discretion. All treatments were continued until disease progression, intolerable toxicity, or a decision by the physician or participant, whichever occurred first.

In this study, patients with R/M HNSCC harboring Amp11q13 were included as a retrospective external control cohort, receiving PD-1 inhibitors with or without chemotherapy as the first-line therapy. This cohort was incorporated to enable further analysis of the objective response rate (ORR) and survival outcomes. Prior findings from our research indicate that patients with Amp11q13 are unlikely to derive clinical benefit from anti-PD-1 therapy (14). Consequently, ethical considerations regarding patient benefit precluded the inclusion of an anti-PD-1 therapy arm in the prospective cohort for this subgroup.

### NGS of clinical samples

Genomic DNA was isolated from FFPE tissue sections using the QIAgen DNA FFPE Tissue Kit (Germantown, MD, USA) for targeted sequencing with a cancer-related gene panel (Genecast Biotech, Wuxi, China). Peripheral blood samples were collected from patients, and genomic DNA was extracted using the Qiagen DNA Blood Mini Kit as a matched control. Library construction was performed using 300 ng of genomic DNA from each participant. Fragment libraries were prepared from sonicated samples, and target regions were enriched using customized IDT library preparation kits (Integrated DNA Technologies, Coralville, Iowa, USA). The captured DNA was amplified, and the paired-end library was sequenced on a NovaSeq 6000 platform (Illumina, San Diego, CA, USA). Bioinformatic analysis was conducted using an in-house program (Genecast Biotech Co., Wuxi, China).

### Treatment assessments

The baseline characteristics included age, sex, primary tumor location, recurrence/metastatic status, and p16 status of the oropharyngeal cancers. Patients were required to undergo baseline computed tomography (CT) scans or magnetic resonance imaging (MRI) for an assessment of measurable disease. programmed death-ligand 1 (PD-L1) expression was determined using the tumor CPS, which represents the number of PD­L1­positive cells divided by the total number of tumor cells × 100.

The primary endpoint was the ORR, which was defined as the proportion of patients exhibiting complete response (CR) or partial response (PR). Clinical responses were categorized as CR, PR, stable disease (SD), or progressive disease (PD) according to RECIST v1.1 criteria. These assessments were conducted using standard clinical practice methods, including CT, MRI, ultrasound, and other imaging modalities. The response rates were calculated based on patients who underwent at least one tumor assessment during the follow-up.

Follow-up and tumor imaging were performed according to the institutional protocols. Assessments were conducted at baseline and every 6 weeks (two cycles) during the first 6 months. If a PR was achieved, assessments were conducted every four cycles. Second-line therapies were documented if administered. Treatment duration was defined as the period from the index date to permanent discontinuation of treatment for any reason, including death. Time-to-event analysis was conducted on the treatment duration to account for any events occurring after treatment initiation that led to treatment interruption.

### Statistical analysis

Quantitative data were summarized using appropriate descriptive statistics, such as the mean, median, and standard deviation. Categorical data, including ORR, are presented as frequencies and percentages. The Kaplan–Meier method was used to calculate OS and PFS, while log-rank tests were used to compare survival curves and assess differences in OS and PFS between the groups. A Cox proportional hazards regression model was used to compare factors with prognostic potential. For categorical data, comparisons between groups were performed using Fisher’s exact or chi-squared tests, as appropriate. The significance level for two-sided p-values was set at 0.05. All statistical analyses were performed using IBM SPSS Statistics software (version 22).

## Results

### Patients

**Figure 1:**
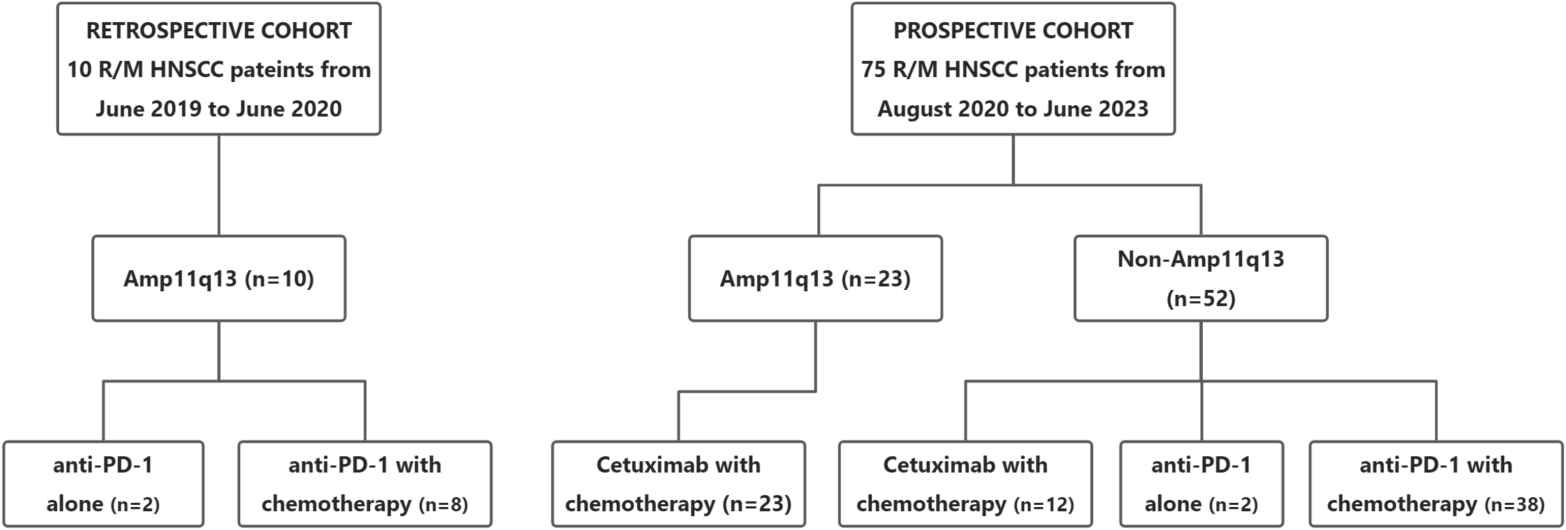
Cohort diagram of first-line treatment according to 11q13 status. R/M HNSCC, recurrent or metastasitc head and neck cancer; Amp11q13, 11q13 amplification; Anti-PD-1, Anti-programmed cell death protein 1.

Between August 2020 and June 2023, a total of 75 patients were enrolled in this prospective study. The predominant primary tumor site was the oral cavity (88.0%). In the prospective cohort, 61 patients (81.3%) presented with unresectable recurrent tumors, six patients (8.0%) exhibited distant metastases, and eight patients (10.7%) had both recurrent tumors and distant metastases. Additionally, ten patients with Amp11q13 were included as external controls in a retrospective cohort between June 2019 and June 2020. The baseline characteristics of the study population are presented in **Table 1**. The details of the treatment regimens are presented in **Figure 1**.

**Table 1:** Baseline Characteristics.

Characteristics	Retrospective cohort (n = 10)	Prospective cohort (n = 75)	
	Amp11q13 (n = 10)	Amp11q13 (n = 23)	non-Amp11q13 (n = 52)
Age Median (range) (yr)	56 (36-65)	58 (28-75)	58 (25-76)
Sex (%)			
Male	10 (100.0)	16 (69.6)	27 (51.9)
Female	0 (0)	7 (30.4)	25 (48.1)
Primary Site (%)			
Oral Cavity	8 (80.0)	19 (82.6)	47 (90.4)
Oropharynx (p16-)	1 (10.0)	3 (13.1)	1 (1.9)
Oropharynx (p16+)	0 (0)	0 (0)	1 (1.9)
Hypopharynx	1 (10.0)	0 (0)	2 (3.8)
Larynx	0 (0)	1 (4.3)	1 (1.9)
Disease Status (%)			
Recurrent	10 (100.0)	16 (69.6)	45 (86.5)
Metastatic	0 (0)	3 (13.0)	3 (5.8)
Recurrent and Metastatic	0 (0)	4 (17.4)	4 (7.7)
Treatment (%)			
Anti-PD-1 w/wo chemo	10 (100.0)	0 (0)	40 (76.9)
Cetuximab with chemo	0 (0)	23 (100.0)	12 (23.1)
CPS (%)			
<1	4 (40.0)	4 (17.4)	8 (15.4)
1-20	3 (30.0)	14 (60.9)	24 (46.2)
≥20	3 (30.0)	5 (21.7)	20 (38.4)

W/wo chemo, with or without chemotherapy; CPS, combined positive score; Amp11q13, 11q13 amplification.

### Treatment and outcomes

In the retrospective cohort, ten patients with Amp11q13 received anti-PD-1Ab with or without chemotherapy (Amp11q13-anti-PD-1Ab group), resulting in an ORR of only 10.0% (**Table 2**). One patient in this group experienced HPD. Patients with Amp11q13 treated with anti-PD-1Ab-based therapy achieved a median PFS of 2.1 months (95% CI: 1.1–3.2) and a median OS of 9.0 months (95% CI: 5.0–13.0) (**Table 2**). For patients treated with an anti-PD-1Ab-based regimen, there were significant differences in median PFS (14.3 months vs. 2.1 months, p<0.001) and OS (38.2 months vs. 9.0 months, p = 0.011) (**Table 2**) between non-Amp11q13 and Amp11q13 patients.

**Table 2:** ORR, PFS and OS in Different Cohorts.

Characteristics	Retrospective cohort (n = 10)	Prospective cohort (n = 75)		
	Amp11q13-anti-PD-1Ab (n = 10)	Non-Amp11q13-anti-PD-1Ab (n = 40)	Non-Amp11q13-Cetuximab (n = 12)	Amp11q13-Cetuximab (n = 23)
ORR (%)	10.0	72.5	75.0	69.6
CR (%)	0	11	0	1
PR (%)	1	18	9	15
SD (%)	3	8	3	5
PD (%)	6	3	0	2
PFS				
6-mon PFS rate (%)	0.0	66.8	50.0	43.5
1-yr PFS rate (%)	0.0	53.4	16.7	19.6
2-yr PFS rate (%)	0.0	38.2	0.0	6.5
Median PFS (months)	2.1	14.3	6.0	5.9
OS				
1-yr OS rate (%)	43.8	78.9	66.7	55.2
2-yr OS rate (%)	14.6	51.3	29.2	30.7
Median OS (months)	9.0	38.2	13.5	16.7

ORR, objective response rate; CR, complete response; PR, partial response; SD, stable disease; PD, progressive disease; PFS, progression-free survival; OS, overall survival.

In the prospective study, 75 patients were enrolled. Of these, 52 patients did not harbor Amp11q13. Forty patients underwent anti-PD-1Ab-based first-line treatment (non-Amp11q13-anti-PD-1Ab group), with 38 receiving anti-PD-1Ab therapy along with chemotherapy and two patients receiving anti-PD-1Ab monotherapy. The confirmed ORR was 72.5% in the non-Amp11q13-anti-PD-1Ab group. Among the 38 patients anti-PD-1Ab with chemotherapy, the ORR was 71.1%, while patients treated with anti-PD-1Ab monotherapy exhibited an ORR of 100%. Twelve patients received cetuximab along with a chemotherapy regimen (non-Amp11q13-cetuximab group), resulting in an ORR of 75.0%. All 23 patients with Amp11q13 received cetuximab along with chemotherapy (Amp11q13-cetuximab group), yielding an ORR of 69.6%. The ORR details for the different groups are listed in **Table 2**. A significant difference in ORR was observed between the non-Amp11q13-anti-PD-1Ab and Amp11q13-anti-PD-1Ab groups (p = 0.0015).

**Figure 2:**
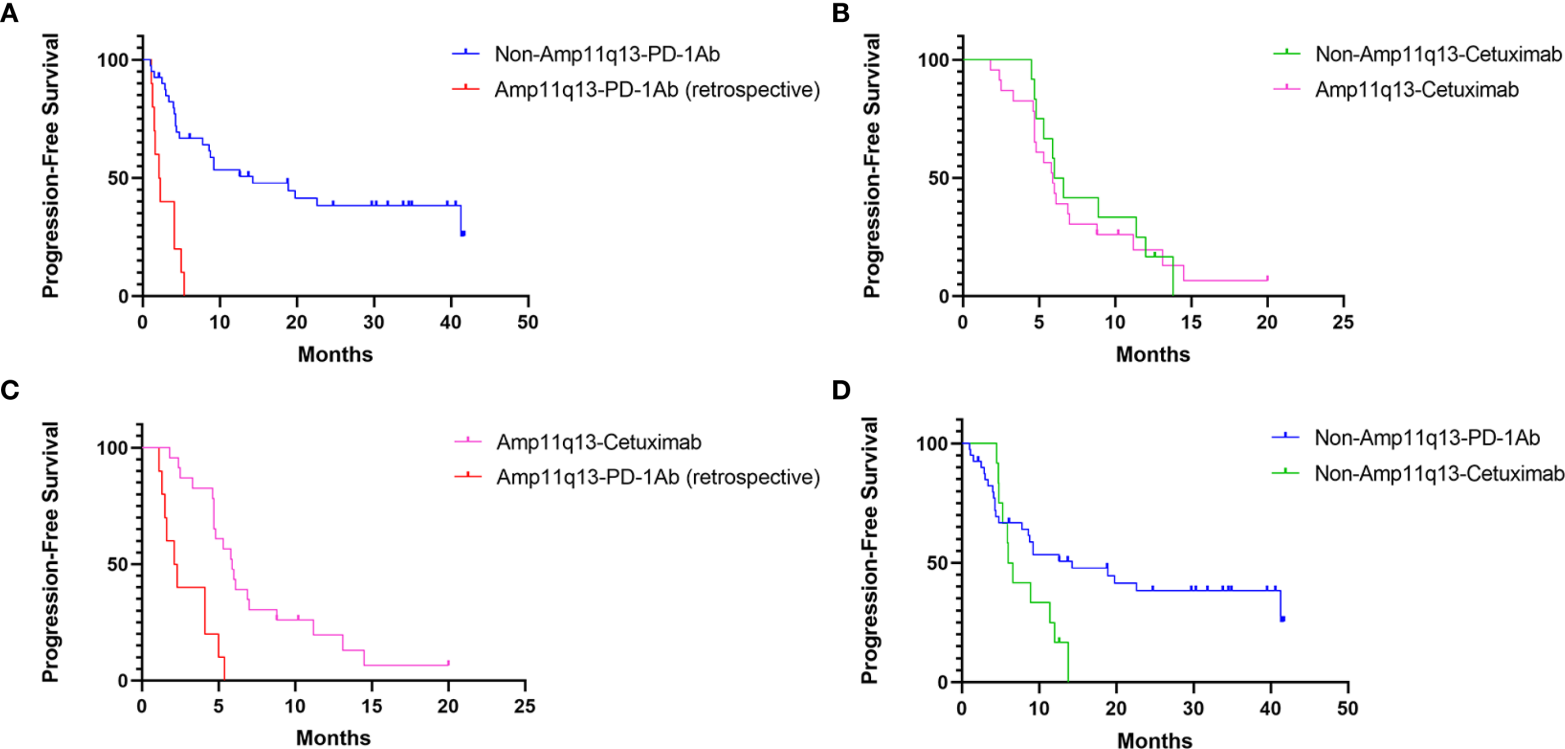
PFS of Different Cohorts: **(A)** PFS of patients receiving PD-1Ab with or without chemotherapy in patients with/without 11q13 amplification (p<0.001). **(B)** PFS of patients’ receiving cetuximab with chemotherapy in patients with/without 11q13 amplification (p=0.750). **(C)** PFS of patients with Amp11q13 receiving different first-line treatments (p<0.001). **(D)** PFS of non-Amp 11q13 patients’ receiving different first-line treatments (p=0.053).

**Figure 3:**
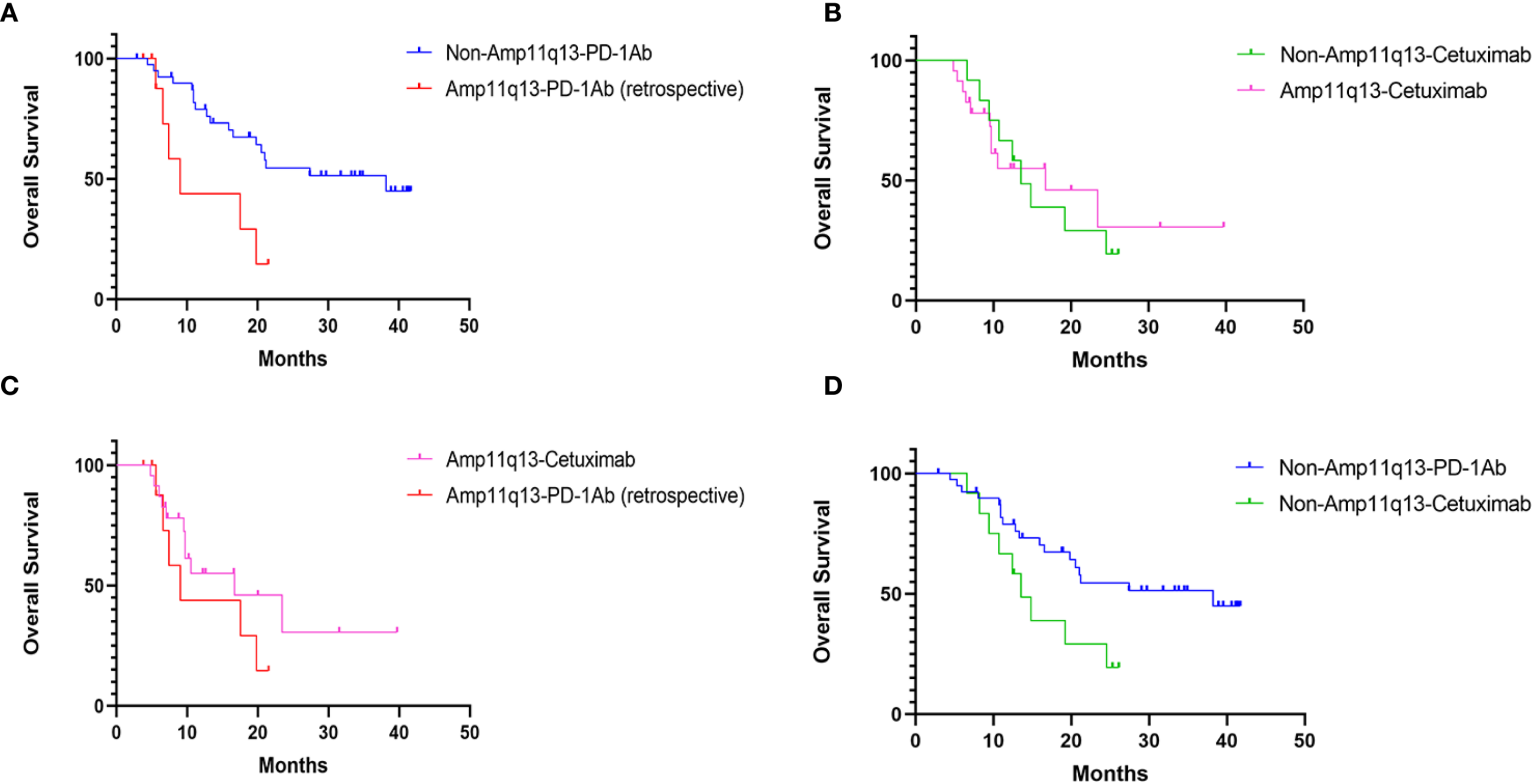
OS in Different Cohorts: **(A)** OS of patients’ receiving PD-1Ab with or without chemotherapy in patients with/without 11q13 amplification (p=0.011). **(B)** OS of patients’ receiving cetuximab with chemotherapy in patients with/without 11q13 amplification (p=0.836). **(C)** OS of patients with Amp11q13 receiving different first-line treatments (p=0.239). **(D)** OS of patients with non-Amp11q13 receiving different first-line treatments (p=0.003).

The data cut-off for the prospective investigation was April 30, 2024. In this prospective cohort study, the median follow-up time was 29.7 months. The median PFS was 14.3 months (95% CI: 2.9–25.7) in the non-Amp11q13-anti-PD-1Ab group, 6.0 months (95% CI: 4.8–7.2) in the non-Amp11q13-cetuximab group and 5.9 months (95% CI: 4.8–7.0) in the Amp11q13-cetuximab group (**Table 2**). The median OS was 38.2 months (95% CI: 12.9–63.5) in the non-Amp11q13-anti-PD-1Ab group, 13.5 months (95% CI: 9.9–17.1) in the non-Amp11q13-cetuximab group, and 16.7 months (95% CI: 3.6–29.8) in the Amp11q13-cetuximab group (**Table 2**). The survival outcomes in the different groups are presented in **Table 2**. Among patients treated with anti-PD-1Ab, significant differences in PFS and OS were observed between the non-Amp11q13 and Amp11q13 groups (**Figures 2A**, **3A**). In patients without Amp11q13, anti-PD-1Ab therapy was associated with a statistically significant improvement in OS (**Figure 3D**). PFS and OS curves for each group are shown in **Figures 2** and **3**, respectively.

In the non-Amp11q13-anti-PD-1Ab group, three patients underwent salvage surgery after achieving a major PR and subsequently continued anti-PD-1Ab monotherapy. At the last follow-up, none of the three patients experienced tumor progression. Among the 24 patients who experienced tumor progression, 16 patients received the second-line treatment, while the subsequent treatment of two patients was unknown due to thexloss of follow-up. Six patients did not receive the second-line treatment primarily because of their deteriorating physical performance.

**Figure 4:**
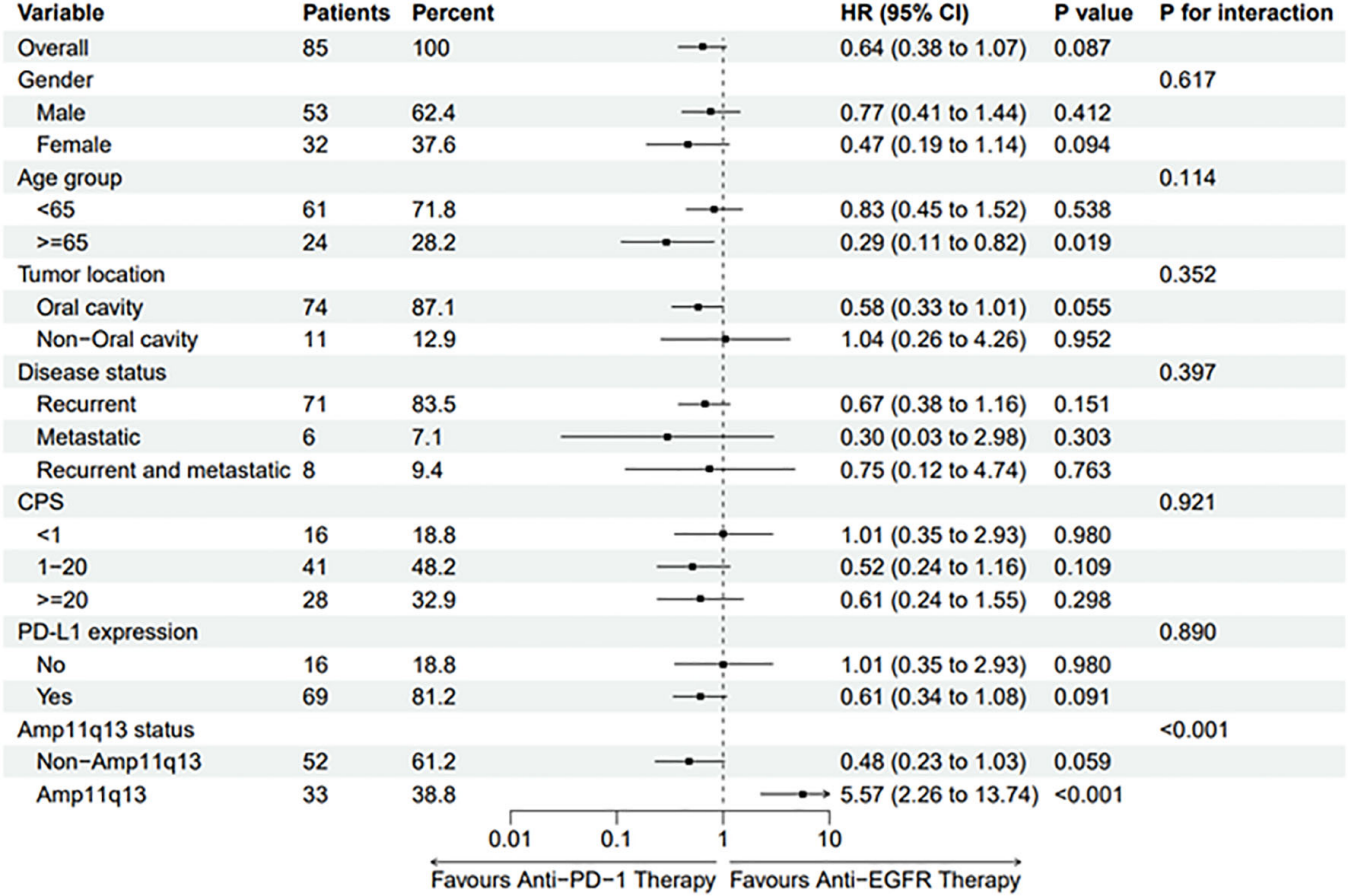
Forest Plot of PFS in Different Subgroups.

Subgroup analysis revealed that the hazard ratio (HR) of PFS for anti-PD-1 therapy compared to cetuximab-chemotherapy was 5.57 (95% CI, 2.26–13.74, p <0.001) among patients with Amp11q13, suggesting that patients with Amp11q13 may not benefit from anti-PD-1 therapy (**Figure 4**). A trend was observed indicating that non-Amp11q13 patients may be more likely to benefit from anti-PD-1 therapy (HR 0.48, 95% CI: 0.23–1.03). Subgroup analyses of CPS and PD-L1 expression showed no statistically significant differences between the two first-line treatment strategies (**Figure 4**).

### Treatment compliance and adverse events

The adverse events (AEs) observed were consistent with those reported in previous literature (3, 4). The treatment-related AEs of grades 3–4 are listed in **Supplementary Table 1**. Two patients discontinued cisplatin due to renal dysfunction, while four others experienced chemotherapy interruptions due to anemia. Additionally, four patients interrupted the treatment due to pneumonia.

Among patients receiving anti-PD-1Ab therapy and chemotherapy, three developed pneumonitis. One case was considered potentially related to the PD-1 inhibitor, resulting in treatment interruption. The remaining two patients were diagnosed with infectious pneumonia.

## Discussion

Our exploratory study evaluated Amp11q13 as a potential predictive biomarker to inform first-line treatment decisions regarding anti-PD-1 therapy in patients with R/M HNSCC. Patients without Amp11q13 who received anti-PD-1 therapy (either as monotherapy or in combination) yielded improved ORR and survival outcomes than other published results. These findings demonstrate the potential of Amp11q13 as a predictive biomarker to optimize immunotherapy in clinics, independent of CPS. Our study is among the first to prospectively use Amp11q13 as a biomarker to guide first-line immunotherapy decisions for R/M HNSCC.

Amp11q13 is a relatively common genetic aberration in HNSCC (9, 12, 13, 17). Our study demonstrated better response to anti-PD-1 treatment in R/M HNSCC patients without 11q13 amplification, providing insights into the application of immunotherapy in real-world settings. Our findings suggest that considering the Amp11q13 status could facilitate the stratification of R/M HNSCC patients who are more likely to benefit from immunotherapy. By excluding patients with Amp11q13 to receive immunotherapy, we could potentially improve treatment outcomes. Underlying mechanisms of Amp11q13 were not assessed in this study. Previous studies in solid tumors have suggested that tumors with *CCND1* amplification may impede immune cells and have an immunosuppressive tumor microenvironment through mechanisms such as immune cell exclusion and exhaustion (15). Moreover, coamplification of the CCND1-FGF locus has been implicated in attenuating antitumor immune responses by establishing an immunosuppressive microenvironment (18). These findings indicate that Amp11q13 likely plays an important role in anti-PD-1 resistance. Translational work in our team is trying to elucidate these mechanisms.

PD-1 inhibitors have significantly improved the management of R/M HNSCC, revealing a subset of patients who may achieve prolonged survival (4). However, the use of PD-1 inhibitors, with or without chemotherapy, as a first-line treatment in the real-world practice requires cautious consideration. Recent studies suggest that not all patients are suitable for first-line immunotherapy (8, 19). Subgroup analyses of the KEYNOTE-048 trial indicated that even patients with a high CPS may not benefit from PD-1 inhibitors and may experience early progression. As shown in the KEYNOTE-048, the rates of PD were 32% (CPS ≥ 20) and 39% (CPS ≥ 1) in the pembrolizumab monotherapy group compared to 10-13% in patients treated with EXTREME regimen (4). Moreover, studies have identified HPD as a concerning outcome in approximately one-quarter or more of patients receiving immunotherapy, particularly those with bulky primary disease (8, 20, 21). These findings underscore the need for more biomarkers to identify appropriate candidates for immunotherapy and to optimize first-line treatment decisions in patients with R/M HNSCC.

In this study, R/M HNSCC patients without Amp11q13 who received anti-PD-1-based therapy exhibited an ORR of 72.5%, a median PFS of 14.3 months, and an OS of 38.2 months. These outcomes appear far more favorable compared to the outcomes of patients in any category reported in the KEYNOTE-048 trial, where the best median PFS was 5.8 months and OS was 14.7 months in the PD-L1 CPS ≥ 20 population treated with pembrolizumab and chemotherapy (4). The phase IV KEYNOTE-B10 study, which included 101 patients with R/M HNSCC treated with pembrolizumab, carboplatin, and paclitaxel, reported an ORR of 48.5% and median PFS of 5.6 months, despite 80.2% of patients having a CPS ≥ 1 (22). In another study, a phase II trial involving 67 Chinese patients with R/M HNSCC demonstrated an ORR of 62.7% and a median PFS of 11.6 months (12). Our study primarily enrolled patients with unresectable recurrent oral squamous cavity carcinoma (OSCC). In the KEYNOTE-048 subgroup analyses, patients with recurrent OSCC treated with pembrolizumab plus chemotherapy showed no benefit compared with the EXTREME regimen (4). Furthermore, younger patients with OSCC or prior treatment with locoregional radiotherapy could be regarded as potential risk factors for HPD among patients with R/M HNSCC treated with ICIs (23). Our findings suggest that patients lacking 11q13 amplification are more likely to benefit from anti-PD-1 therapy, probably associated with a reduced risk of PD. These findings indicate that Amp11q13 status may serve as a predictive biomarker for guiding first-line treatment decisions in R/M HNSCC.

In an externally controlled cohort of ten R/M HNSCC patients with Amp11q13, anti-PD-1 therapy yielded poor ORR and PFS. This observation further strengthens the correlation between Amp11q13 and reduced efficacy of anti-PD-1 therapy in patients with R/M HNSCC, irrespective of CPS status (14). Singavi et al. reported that patients harboring chromosome Amp11q13 exhibited disease progression during the initial treatment, with a 43% incidence of hyperprogression (24). A recent study also associated Amp11q13 with poor ORR in R/M HNSCC (12).

Recently, the KEYNOTE-689 trial investigated the incorporation of neoadjuvant and adjuvant pembrolizumab (anti-PD-1) into standard care for patients with locally advanced HNSCC (LA-HNSCC) (25). Despite its potential, disease progression was observed in a subset of patients during neoadjuvant pembrolizumab treatment. The treatment failure for this subgroup might be due to the 11q13 amplification. Our findings suggest that patients lacking 11q13 amplification are more likely to exhibit a favorable response to anti-PD-1 therapy, indicating that 11q13 amplification status may inform clinical decision-making regarding the utilization of immunotherapy in HNSCC. While incorporating 11q13 amplification testing into clinical decision-making may enhance personalized treatment strategies for HNSCC, careful consideration is warranted to ensure its application. Due to the lack of universal standard for defining gene amplifications and sensitivity of NGS to detect large-scale chromosomal aberrations, it would be better to validate 11q13 amplification by fluorescence *in situ* hybridization (FISH) or array comparative genomic hybridization (array CGH). The potential interaction between 11q13 amplification and other biomarkers, such as CDKN2A, was not included in the design or analysis of this study. Existing literature suggests that CDKN2A alterations may influence immunotherapy response in HNSCC (26). Further studies are needed to explore their potential interactions.

Despite the valuable information provided by our investigation, our results should be interpretated cautiously due to the limitations. A key limitation is the inability to conduct a randomized controlled trial. Due to the ethical concern, as prior evidence suggests limited benefit of anti-PD-1 therapy in Amp11q13 patients (14). Instead, we used historical data from Amp11q13 patients treated with anti-PD-1 therapy as a control cohort, preserving ethical standards and enabling comparison. However, the retrospective cohort’s small sample size and retrospective nature limited its statistical power. Additionally, this study did not explore mechanisms of immunotherapy resistance in Amp11q13 patients. Future research should investigate these mechanisms to enhance treatment stratification. Furthermore, considering that our study primarily included patients with OSCC, further research is required to validate the role of 11q13 amplification and its potential as a biomarker across other HNSCC subtypes such as oropharyngeal or laryngeal cancers.

In conclusion, our study highlights the potential of Amp11q13 as a predictive biomarker for immunotherapy in the first-line treatment of R/M HNSCC. These findings offer valuable insights for optimizing the immunotherapy as the first-line treatment decisions for R/M HNSCC, although further validation is needed.

## Data Availability

The data supporting the conclusions of this study are available upon reasonable request to the corresponding author.
