# Supplementary material for: Chromosome 11q13 amplification as a decision-making biomarker for anti-PD-1 immunotherapy in recurrent or metastatic head and neck squamous cell carcinoma: a prospective cohort study
Source: Front Immunol. 2025 Oct 13;16:1667733. doi: 10.3389/fimmu.2025.1667733 (PMC12554652; doi:10.3389/fimmu.2025.1667733)
Supplement: Supplementary file 1 [file Table1.docx]

Supplemental Table 1. Treatment-Related Adverse Events of Grade 3-4.

|  | Amp11q13-Cetuximab (n = 23) | Non-Amp11q13-PD-1Ab (n = 40) | Non-Amp11q13-Cetuximab (n = 12) |
| --- | --- | --- | --- |
| Neutropenia | 3 (13.0) | 7 (17.5) | 1 (8.3) |
| Anemia | 0 (0) | 3 (7.5) | 1 (8.3) |
| Fatigue | 1 (4.3) | 2 (5.0) | 0 (0) |
| Infectious pneumonitis | 1 (4.3) | 2 (5.0) | 0 (0) |
| Immune-related pneumonitis | 0 (0) | 1 (2.5) | 0 (0) |
| Rash | 1 (4.3) | 0 (0) | 0 (0) |
